# Optimizing the identification of risk‐relevant mutations by multigene panel testing in selected hereditary breast/ovarian cancer families

**DOI:** 10.1002/cam4.1251

**Published:** 2017-12-22

**Authors:** Anna Coppa, Arianna Nicolussi, Sonia D'Inzeo, Carlo Capalbo, Francesca Belardinilli, Valeria Colicchia, Marialaura Petroni, Massimo Zani, Sergio Ferraro, Christian Rinaldi, Amelia Buffone, Armando Bartolazzi, Isabella Screpanti, Laura Ottini, Giuseppe Giannini

**Affiliations:** ^1^ Department of Experimental Medicine University La Sapienza V.le R. Elena 324 Rome 00161 Italy; ^2^ Department of Molecular Medicine University La Sapienza V.le R. Elena 291 Rome 00161 Italy; ^3^ Center for Life Nano Science@Sapienza Istituto Italiano di Tecnologia Rome 00161 Italy; ^4^ Department of Pathology Sant'Andrea Hospital University La Sapienza Via di Grottarossa 1035 Rome 00189 Italy; ^5^ Istituto Pasteur‐Fondazione Cenci Bolognetti Rome 00161 Italy

**Keywords:** *ATM*, BRCAPro5, *CHEK2*, hereditary breast cancer, NGS

## Abstract

The introduction of multigene panel testing for hereditary breast/ovarian cancer screening has greatly improved efficiency, speed, and costs. However, its clinical utility is still debated, mostly due to the lack of conclusive evidences on the impact of newly discovered genetic variants on cancer risk and lack of evidence‐based guidelines for the clinical management of their carriers. In this pilot study, we aimed to test whether a systematic and multiparametric characterization of newly discovered mutations could enhance the clinical utility of multigene panel sequencing. Out of a pool of 367 breast/ovarian cancer families Sanger‐sequenced for *BRCA1* and *BRCA2* gene mutations, we selected a cohort of 20 *BRCA1/2*‐negative families to be subjected to the BROCA‐Cancer Risk Panel massive parallel sequencing. As a strategy for the systematic characterization of newly discovered genetic variants, we collected blood and cancer tissue samples and established lymphoblastoid cell lines from all available individuals in these families, to perform segregation analysis, loss‐of‐heterozygosity and further molecular studies. We identified loss‐of‐function mutations in 6 out 20 high‐risk families, 5 of which occurred on *BRCA1*,*CHEK2* and *ATM* and are esteemed to be risk‐relevant. In contrast, a novel *RAD50* truncating mutation is most likely unrelated to breast cancer. Our data suggest that integrating multigene panel testing with a pre‐organized, multiparametric characterization of newly discovered genetic variants improves the identification of risk‐relevant alleles impacting on the clinical management of their carriers.

## Introduction

About 5–10% of breast and/or ovarian cancer cases have a hereditary background, mainly dependent on highly penetrant mutations in the *BRCA1* and *BRCA2* genes 1. The reported cumulative breast cancer risk by the age of 70 is 55–65% for *BRCA1* and 45–47% for *BRCA2* mutation carriers, while the ovarian cancer risk is 39% for *BRCA1* and 11–17% for *BRCA2* mutation carriers 2, 3. Differences in mutation type and site may at least partially impact on cancer risk definition 4, 5.

*BRCA1* and *BRCA2* gene mutations are typically found in 25–30% of the breast cancer families subjected to genetic testing 6, 7. Therefore*,* the search for germline mutations has often remained negative even in families with a Mendelian inheritance pattern for breast and/or ovarian cancer 8. However, recent improvements in DNA sequencing technology enabled massively parallel sequencing of multiple targets, dramatically improving the speed and the efficiency of DNA testing. A number of different multigene panels have been designed for the analysis of hereditary cancer syndrome families, which may include relatively few and syndrome‐oriented target genes, or much larger gene sets 9, 10, 11. Similar approaches have now been applied to the screening of large cohorts of *BRCA1/2* negative hereditary breast/ovarian cancer families finding mutations in non‐*BRCA1/2* genes in 4–11% of the cases, depending on the features of the patients cohorts and/or on the size of the multigene panel 10, 11, 12, 13, 14, 15. In these studies, loss‐of‐function mutations were identified either in known, syndrome‐related highly penetrant genes (i.e., *TP53* and *PTEN*) or in a number of rarely mutated targets, whose impact on breast/ovarian cancer risk is largely undefined 11, 15, 16. Accordingly, the introduction of Next generation sequencing (NGS) multigene panels for diagnostic purposes is still debated, since their clinical utility is often limited by scant information on the cancer risk conferred by rare genetic variants and lack of evidence‐based guidelines for the clinical management of their carriers 16. Large case–control studies have recently confirmed *PALB2* as a high‐risk breast cancer gene, but they still reached conflicting results on *ATM* and *CHEK2* and rejected the role of many other genes, such as those of the MRN complex 17, 18.

Defining the impact of gene mutations on cancer risk might be a very difficult task especially for rarely mutated genes, hit by different mutation types in different sites. Nonetheless, aiming at this target is mandatory for a correct application of multigene panel sequencing in the clinical settings. To focus on this key question, we reasoned that integrating gene panel sequencing with the systematic use of prediction tools, cosegregation and loss of heterozygosity (LOH) analysis, together with the availability of patient‐derived lymphoblastoid cell lines (LCL) for functional studies could provide significant hints on the impact of newly discovered gene mutations on cancer risk, improving the clinical utility of NGS screenings. In this pilot study, we report on the application of this strategy to a cohort of twenty breast/ovarian cancer families with a moderate‐to‐high probability to be mutation carriers.

## Materials and Methods

### Family recruitment

About 367 breast and/or ovarian cancer families were enrolled at the Hereditary Tumors section of the Policlinico Umberto I, University La Sapienza, and probands have been subjected to *BRCA1/2* mutation screening (Table S1) 19, 20. Out of the *BRCA1/2* negative group, we selected 20 (BRCAX) families characterized by high probability to be mutation carriers, as described in the Results section. DNA samples from peripheral blood or cancer tissues, were subjected to multigene panel NGS or Sanger sequencing, respectively. Lymphoblastoid cell lines (LCL) were generated from probands and available relatives, using a standard protocol 21. A careful pretest counseling has been offered to all probands and their relatives to obtain a truly informed consent. All investigations were conducted according to the principles outlined in the declaration of Helsinki.

### *BRCA1/2* mutation screening

Genomic DNA was extracted from peripheral blood samples using a commercial kit (QIAamp Blood Kit, Qiagen, Valencia, CA). The entire coding sequence and all intron/exon boundaries of *BRCA1* and *BRCA2* were screened by direct sequencing using an ABI PRISM DyeDeoxy Terminator Cycle Sequencing Kit and an ABI 3130XL Genetic Analyzer (Applied Biosystems, Warrington, UK) as previously described 19, 20. Sequences were compared against *BRCA1* and *BRCA2* reference sequences (GenBank NM_007294.3 and NM_000059.3; additional GenBank reference sequence were as follows: *ATM*, NM_000051.3; *CHEK2,* NM_007194.3; *RAD50,* NM_005732.3). DNA mutation nomenclature followed current guidelines of the Human Genome Variation Society (http://www.hgvs.org/rec.html). *BRCA1/2* genomic rearrangements were searched for by the Multiple‐Ligation‐dependent‐Probe‐Amplification (MLPA) methodology according to the manufacturer's instructions (MRC–Holland, Amsterdam, the Netherlands) and as described 22.

### Next generation sequencing

NGS and data analysis have been performed on service at the University of Washington‐Seattle using the BROCA‐Cancer Risk panel (http://tests.labmed.washington.edu/BROCA), an approach of genomic capture and massively parallel sequencing of 41 putative breast/ovarian cancer genes, according to Walsh et al. 12. All described variants were subsequently validated in our laboratory by Sanger sequencing.

### RNA extraction and RT‐PCR

LCLs were untreated and treated with cycloheximide (CHX, 100 *μ*g/mL) for 4 h in order to block non‐sense mediated decay. Total RNA extraction was performed using TRI Reagent^®^ (Sigma‐Aldrich, Co.) according to the manufacturer's instructions. 1 *μ*g of the RNA was retro‐transcribed and PCR amplified as described 23 (primer sequences are available on request).

### Tumor‐tissue histology

For each paraffin‐embedded tumor, six 10‐*μ*m paraffin slides were used for genomic‐DNA isolation 24, 25 and a hematoxylin‐eosin–stained slide was used for histopathological examination.

## Results

On a sample of 266 breast cancer (BC) and 101 breast/ovarian cancer families (BOC) Sanger sequenced for *BRCA1/2* genes, 97 (26%) carried deleterious mutations (Table S1). In line with the literature 1, 7, the mutational rate in BC families was dramatically lower (13%) compared to BOC families (60%), suggesting that a high percentage of them remains without a conclusive genetic diagnosis.

To extend the possibility to identify mutations responsible for breast cancer inheritance, we applied multigene panel sequencing to a small cohort of hereditary BC/BOC families, which shared the following criteria: (1) being negative for *BRCA1/2* truncating or missense deleterious mutation, after standard Sanger sequencing; being willing to provide; (2) blood and DNA samples from affected and unaffected individuals to establish LCLs and to perform segregation analysis; and (3) tumor tissue from at least one affected individual to perform LOH studies. In order to provide a proof‐of‐concept that this strategy may enhance the identification of mutations impacting on cancer risk definition and clinical management of the carrier families, we limited the cohort to 20 families having a clear dominant inheritance pattern and/or a high BRCAPro score. Consistent with the much lower level of *BRCA1/2* mutation rate in BC families, 17 out of 20 were BC families and only three were BOC families (Table 1). Of note, 11 out of the 20 probands showed a very high BRCAPro score (between 97% and 75%) and 6 had a BRCAPro score between 50% and 67%. We also included three families with a BRCAPro <50% that showed co‐occurrence of OC and BC, or the presence of bilateral BC before age 38 years in the proband, plus significant family history (Table 1; Table S2).

**Table 1:** Clinical characteristics of BRCAX Probands

	No. of cases	(%)
Proband cancer history	20	
Unilateral breast	9	45
Bilateral breast	10	50
Ovarian	1	5
Second primary malignancy	2	10
Proband age of cancer onset	20	
25–35	4	20
36–50	14	70
>50	2	10
BRCApro‐5 score (%)	20	
>75	11	55
50–75	6	30
<50	3	15

**Figure 1:**
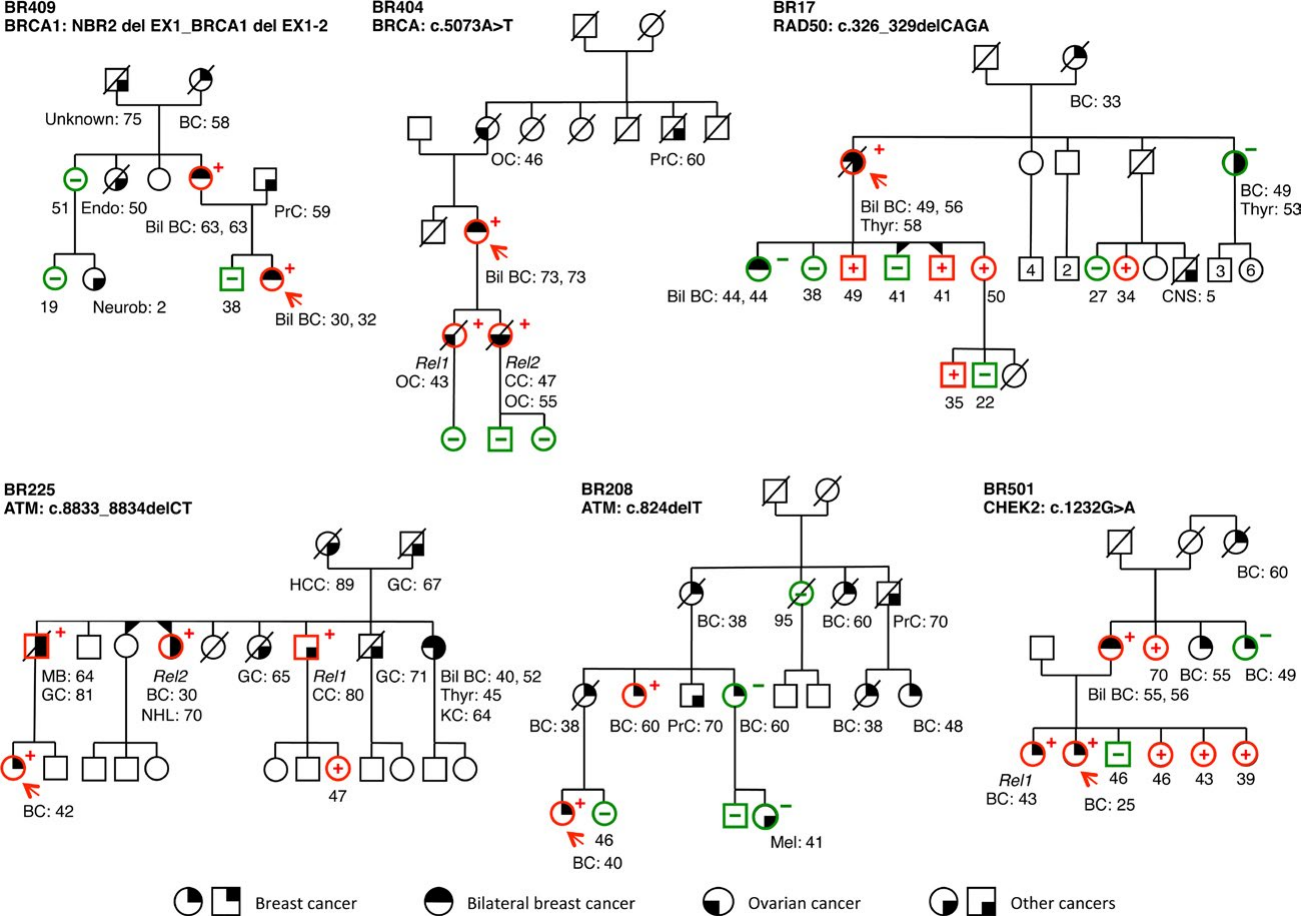
Pedigrees of the six families with germline mutations identified by NGS. Probands are indicated with an arrow. Tested family members are marked with “**+**” for mutation carriers and “**−**” for wild‐type. *Rel1, Rel2:* LOH tested family members. Cancer type and age at diagnosis are reported and described as: BC, breast cancer; bil BC, bilateral breast cancer; OC, ovarian cancer; MB, male breast; PrC, prostatic cancer; Unknown cancer; Neurob, neuroblastoma cancer; NHL, non‐Hodgkin lymphoma; Thyr, Thyroid; KC, Kidney cancer; Mel, melanoma; CNS, central nervous system cancer; GC, gastric cancer; CC, colon cancer; Endo, endometrial cancer.

By this approach, we identified loss‐of‐function mutations in 6/20 (30%) probands, five of which occurred on *BRCA1*,* CHEK2*, and *ATM* and are esteemed to be risk‐relevant according to our studies (Table 2, Fig. 1), while a novel *RAD50* truncating mutation is most likely unrelated to breast cancer. Interestingly, all these mutations occurred in families with a very high BRCAPro score (75–97%).

**Table 2:** Mutations identified by NGS

Family ID	Mutation		BRCAPro	Segregation				Total
				Healthy		Affected		
			%	C	NC	C	NC	
BR409	*BRCA1/NBR2*	NBR2delEX1_BRCA1delEX1‐2	91	0	3	2	0	5
BR404	*BRCA1*	c.5073A>T (p.Thr1691=)	75	0	3	3	0	6
BR225	*ATM*	c.8833_8834delCT (p.Leu2945 fs)	91	1	0	4	0	5
BR208	*ATM*	c.824delT (p.Leu275Ter)	82	0	3	2	2	7
BR501	*CHEK2*	c.1232G>A (p.Trp411Ter)	94	4	1	3	1	9
BR17	*RAD50*	c.326_329delCAGA (p.Thr109 fs)	97	5	4	1	2	12

C, carrier; NC, no carrier.

**Figure 2:**
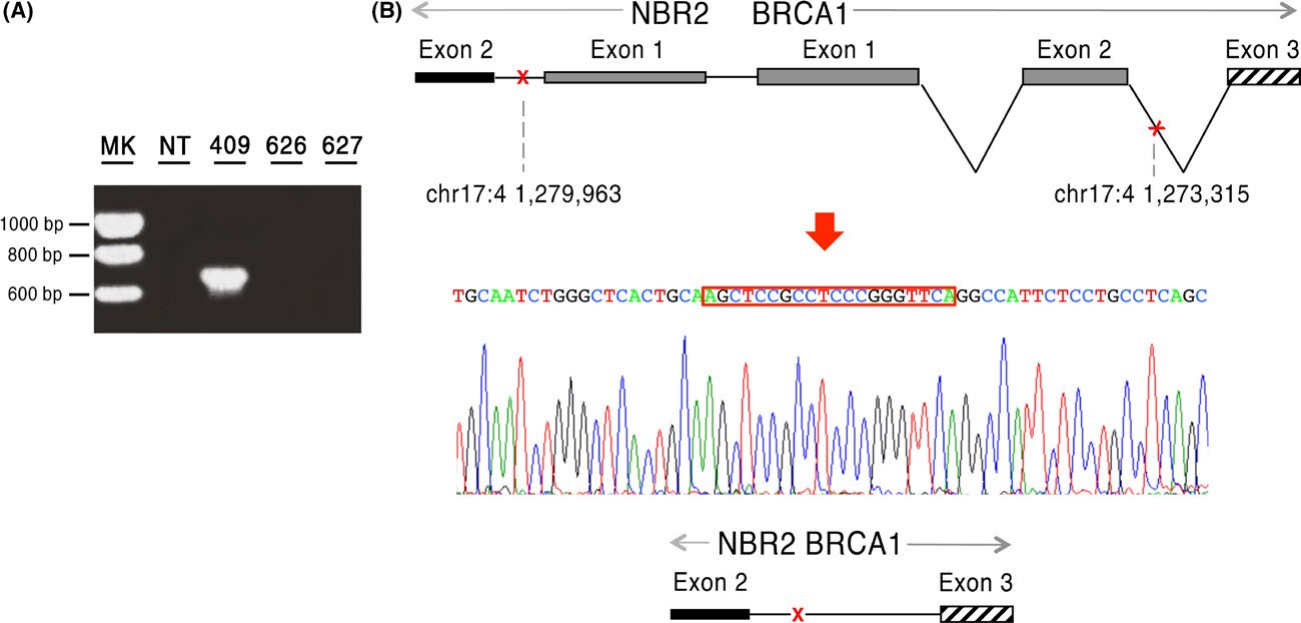
*BRCA1/NBR2* rearrangement identified in the BR409 family. (A) Gel image of PCR products. PCR amplification of the genomic region spanning the *BRCA1/NBR2* rearrangement resulted in a fragment of approximately 670 bp present only in the proband BR409. (B) Schematic representation and electropherogram showing the *NBR2* exon1 and *BRCA1* exons 1 and 2 deletion. The variant arose from an erroneous homologous recombination process between two AluY motifs, localized at chr17:41279963 and at chr17:41273315, respectively, and it involved a perfectly repeated stretch of 20 bp. MK, marker; NT, no template; 409 proband DNA, 627, 626 healthy individual DNA.

Despite previous *BRCA1/2* testing, the NGS approach identified two novel *BRCA1* deleterious mutations. One is a *BRCA1/NBR2* rearrangement (*NBR2*del EX1_*BRCA1* delEX1‐2). PCR amplification of genomic DNA from the BR409 proband resulted in an aberrant fragment of approximately 670 bp (Fig. 2A), whose direct sequencing confirmed the putative breakpoints. As reported in Figure 2, loss of *NBR2* exon1 and *BRCA1* exons 1 and 2 possibly originated from an erroneous homologous recombination process between two AluY motifs, located at chr17:41279963 and at chr17:41273315, respectively (Fig. 2B). We detected this mutation in a 38‐year‐old woman (BR409), with bilateral breast cancer at age 30 and 32, belonging to a very high‐risk family (BRCAPro 91%) (Fig. 1). As expected, this mutation cosegregates with the disease (Table 2).

**Figure 3:**
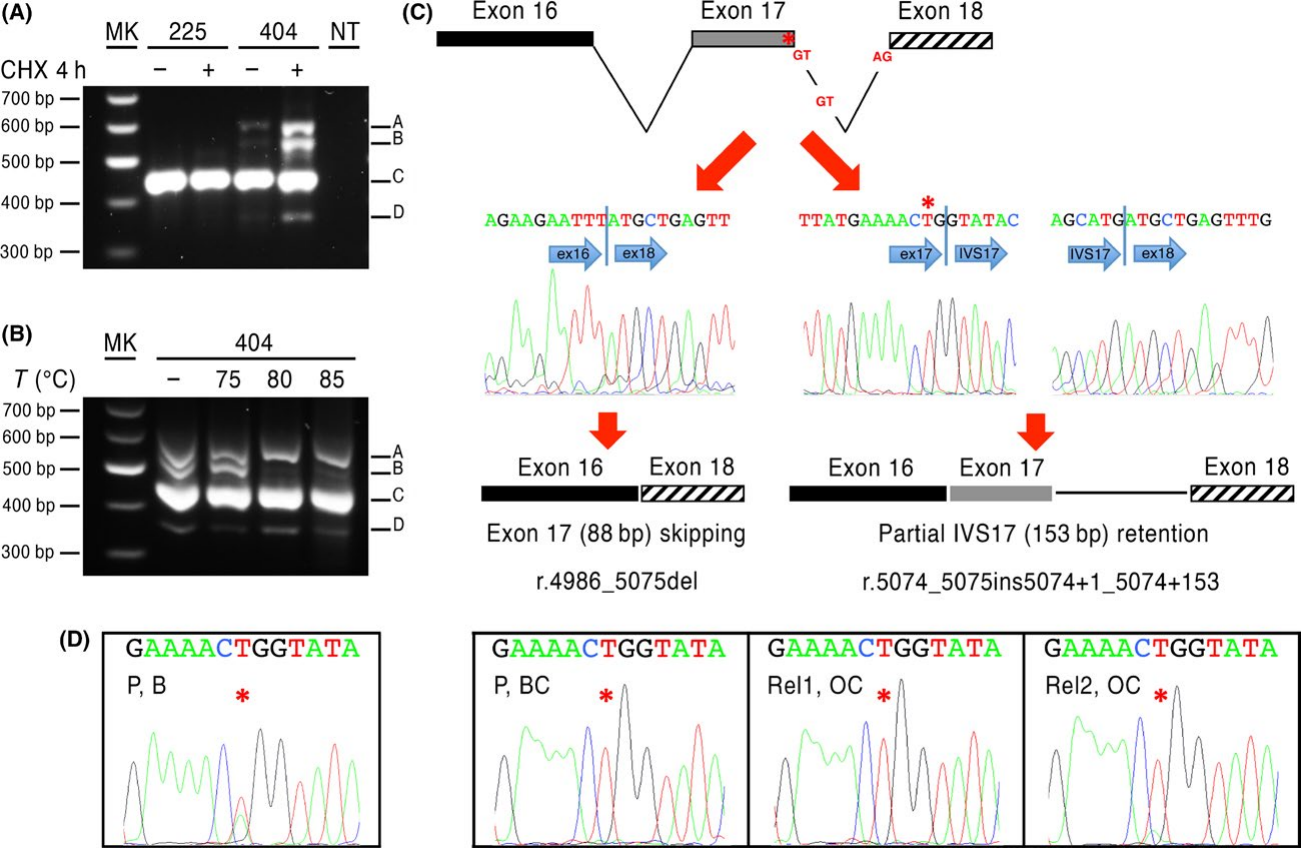
*BRCA1* c.5073A>T (p.Thr1691=) identified in the BR404 family. (A) PCR amplification of the alternative transcripts in patient 404 mRNA from LCL exposed or non‐exposed to cycloheximide (CHX): (A) 604 bp aberrant fragment; (B) 570 bp aberrant fragment; (C) wt transcript fragment and (D) 363 bp aberrant fragment. (B) Melting and reannealing PCR fragments at rising temperatures (80° and 85°C), allow disappearance of band B, which indicates it is a heteroduplex. (C) Schematic representation and electropherograms of the excised bands showing the presence of a transcript lacking exon17 and of an aberrant transcript retaining a 153 bp fragment of intron 17. (D) Electropherograms of DNA obtained from the blood and cancer tissues of proband and her daughters showing LOH with conservation of the c.5073A>T (p.Thr1691=) allele in all cancer tissues (Table S3). MK, marker; NT, no template; 225 control LCL; 404 proband LCL; P, B, Proband blood sample; P, BC, Proband breast cancer tissue; Rel1, OC, Relative 1 ovarian cancer tissue; Rel2, OC, Relative 2 ovarian cancer tissue.

The second *BRCA1* mutation is a synonymous variant on the last codon of exon 17 (c.5073A>T; p.Thr1691=) already identified, but not considered relevant, at the time of the first Sanger sequencing. The NNSPLICE prediction tool (http://fruitfly.org:9005/seq_tools/splice.html) suggested it could cause an alternative splicing with skipping of exon 17, and exon 16–18 out‐of‐frame joining. The functional consequences on this mutation was ascertained by RT‐PCR and sequencing analysis of the transcripts in LCLs, which identified the wild type form (Fig. 3A, fragment C) and the predicted aberrant transcript (Fig. 3A, fragment D) skipping exon 17 and carrying a premature stop codon at residue 1672 (p.Met1663 fs). Moreover, we observed an additional transcript (Fig. 3A, fragment A), incorporating a 153 bp sequence of intron 17 (preceding a typical GT 5′‐splice signal) that created a stop codon at residue 1706 (p.Asp1692fs). A further 570 bp band (Fig. 3A, fragment B), instead, proved to be a heteroduplex rather than a specific splicing product (Fig. 3B). All the aberrant transcripts were detected in the BR404 proband, but not in control LCLs and they increased with cycloheximide (CHX) treatment, suggesting they all suffer a non‐sense mediated decay (Fig. 3A). We identified the c.5073A>T (p.Thr1691=) variant in a woman (BR404, BRCAPro 82%) with bilateral breast cancer at age 73 (Fig. 1). Also in this case, the mutation cosegregated with the diseases in 100% of the cases (Table 2). Moreover, we showed LOH for the mutant allele in the tumor tissue of the proband and both her daughters (Fig. 3D; Table S3). These data strongly suggest that the *BRCA1/NBR2* rearrangement and *BRCA1* c.5073A>T (p.Thr1691=) are new loss‐of‐function and cancer risk‐relevant mutations.

Two protein‐truncating variants occurred on the *ATM* gene. The *ATM* exon 7 c.824delT mutation resulting in a premature termination at codon 275 (p.Leu275Ter) occurred in the BR208 proband (BRCAPro 82%) who was diagnosed with breast cancer at age 40 (Fig. 1). This mutation cosegregated with the disease in two family members affected with early onset breast cancer, but not in an elderly BC case, four unaffected relatives and one melanoma case (Table 2). LOH analysis showed maintenance of a heterozygous state of the *ATM* alleles in normal, preneoplastic and neoplastic tissue from the proband (Table S3).

The *ATM* exon 61 c.8833_8834delCT mutation introduced a stop codon at position 2954 (p.Leu2945fs). It occurred in the BR225 proband (BRCAPro 91%) affected with breast cancer at age 42 (Fig. 1). This mutation cosegregated with the disease in three BC patients (including one male) and one colorectal cancer patients (Table 2). Also in this case, LOH analysis showed maintenance of a heterozygous state, in all tissues examined (Table S3). These results were consistent with a risk‐relevant role of *ATM* mutations in both families.

The *CHEK2* nonsense c.1232G>A mutation on exon 11 resulted in a premature protein termination at codon 411 (p.Trp411Ter) predicted to disrupt protein function and occurred in the BR501 proband (BRCAPro 94%), affected with an early onset breast cancer at age 25 (Fig. 1). Extensive segregation analysis in nine individuals of the family indicated the mutation segregated with most breast cancer cases (three out four) (Table 2). Moreover, we showed LOH for the c.1232G>A mutation in the tumor tissue (Table S3), supporting a risk‐relevant role for this mutation.

The novel *RAD50* frameshift c.326_329delCAGA mutation in the exon 3 introduced a stop codon at position 128 (p.Thr109fs), at the level of the ATPaseN domain, which predicts a very strong impact on the function of the RAD50‐MRE11 complex 26. This mutation occurred in the BR17 proband (BRCAPro 97%), who developed bilateral breast and thyroid cancer at 49, 56, and 58 years, respectively (Fig. 1). Nonetheless, segregation analysis, performed in 12 individuals did not support a critical role of this mutation, since it did not segregate in the other two breast cancer cases (one of which bilateral, at 44) (Table 2).

## Discussion

Recently, the landscape of genetic risk evaluation for breast/ovarian cancer expanded due to the introduction of the NGS technology, which has greatly simplified the search for genetic alterations in targets other than *BRCA1/2*. Many different genes were shown to be mutated in breast/ovarian cancer cohorts, being *PALB2*,* ATM*, and *CHEK2* the most frequent 10, 11, 12, 13, 14, 27. However, the clinical utility of these studies is still controversial since even large case–control studies failed to firmly establish an increased risk for breast and/or ovarian cancer associated with many of the mutated genes 16.

In this pilot study, we reported that association of NGS screenings with the systematic use of prediction tools, cosegregation and LOH analysis and establishment patient‐derived LCL for functional studies improves the identification of risk‐relevant gene mutations. A similar approach had been previously used to establish the clinical significance of uncharacterized BRCA1/2 missense mutations 28. Indeed, the information gathered by this approach influenced the choice for the appropriate risk‐reducing strategy for 5/6 families, in which loss‐of‐function mutations have been detected.

This was rather straightforward for two of them, which are novel *BRCA1* disease causing mutations, falling into class V, according to Plon et al. 29. Indeed, one is a novel *BRCA1/NBR2* rearrangement, which may lead to lack of *BRCA1* transcription, as described for previously reported genomic rearrangements 30, 31. The second is a novel *BRCA1* synonymous variant (c.5073A>T; p.Thr1691=). Our studies on LCL were crucial to demonstrate it gives rise to aberrant alternative transcripts that undergo nonsense‐mediated decay and code for truncated proteins. Since, similar outcomes were previously reported for the c.5074+1G>T variant affecting the consensus GT splicing signal in intron 17 32, our data reveal that the c.5073A>T substitution impairs a strong splicing enhancer. The segregation pattern of both mutations and LOH analysis further support the disease‐causing role of both *BRCA1* mutations.

The two *ATM* protein‐truncating mutations we identified for the first time in breast cancer families, had already been reported as either homozygous or compound heterozygous alterations in Ataxia‐Telangiectasia patients 33, 34, but their impact on cancer risk was not previously described. These mutations segregate in most siblings affected with breast and/or other types of cancer. Interestingly, we observed no LOH for *ATM* variants, in line with the hypothesis that one mutant *ATM* allele may be sufficient to promote tumor initiation 35, 36. Importantly, both *ATM* mutations were also picked up by the p53 mitotic centrosomal localization test, indicating they are functionally impaired in governing p53 centrosomal localization 37. Overall, our data strongly support the role of these *ATM* mutations in cancer development, in these families.

The novel c.1232G>A is a truncating and function disrupting mutation of the *CHEK2* gene, identified in an early onset breast cancer proband. The high number of breast cancers observed in this family, cosegregation of the variant with the disease and its LOH in the breast cancer tissue, strongly suggest this is a breast cancer predisposing allele.

It is worth mentioning that, in principle, the role of mutant *ATM* and *CHEK2* as breast cancer genes is still debated. However, many studies and a large meta‐analysis agree that *ATM* mutations confer a moderate breast cancer risk 36, 38, 39 although there might be differences between truncating versus missense mutations 40. Moreover, a specific missense allele (c.7271T>G) was reported to be associated even with high breast cancer risk 41. In a similar way, the reported breast cancer risk for *CHEK2* mutations varies largely. The cumulative breast cancer risk conferred by the 1100delC *CHEK2* variant is 37% at 70 years 42. Association studies on four different *CHEK2* variants, indicated a breast cancer risk of 20–44% and 9–12% for truncating mutations and missense mutation carriers, respectively, largely depending on their family history 43. A recent case–control study on a large cohort of patients subjected to multigene panel testing further suggest that *ATM* and *CHEK2* are associated with moderate breast cancer risk 18, providing support to NCCN recommendations for an annual magnetic resonance screening of these mutation carriers, starting at age 40 44. Based on our observations and on the knowledge that other genes, such as *PALB2*, have been recently re‐classified from moderate to high‐penetrance BC susceptibility gene 45, we suggest that extreme caution is required in defining *ATM‐* or *CHEK2*‐dependent cancer risk, and that at least extensive segregation analysis and LOH studies should be performed for each mutation, in order to more appropriately define the clinical utility of mutation detection for those genes.

Interestingly, the novel c.326_329delCAGA *RAD50* mutation identified in the BR17 family with a very high BRCAPro score (97%) exemplifies the challenges in transferring genetic data into clinical management deriving from multigene panel sequencing, and further contribute to support the efficacy of our strategy. Indeed, while this protein‐truncating mutation predicts loss‐of‐function of the RAD50‐MRE11 complex 26, it did not cosegregate with breast cancer, suggesting it is unlikely to be the risk‐relevant allele in the BR17 family, where additional genetic alterations might be responsible for cancer inheritance. Importantly, large case–control studies have also indicated lack of significant association between MRN complex gene mutations and breast cancer risk 17, 18.

Although uneasy to be performed on large scale and in non‐research oriented environment, our proposed approach identified risk‐relevant mutations in 25% of the analyzed families. Excluding the two *BRCA1* mutations, we identified risk‐relevant mutations in non‐*BRCA1/2* genes in 17% of the families. Therefore, the combination of multigene panel sequencing with extended and multiparametric characterization of the discovered mutations, eventually restricted to families with high probability of being mutation carriers, stands as very useful approach to increase the clinical utility of NGS screening for inherited breast/ovarian cancer.

Finally, we cannot overlook that a consistent number of high‐risk families still remained without satisfying answers despite multigene panel sequencing, in our and other's studies 13, 46. The overall strategy depicted here could be further exploited to identify risk‐relevant mutations in these families by additional mutation screenings such as whole exome sequencing and/or RNAseq.

In conclusion, we have shown that integrating the systematic use of cosegregation analysis, LOH and functional studies performed on LCL with multigene panel sequencing may improve its clinical utility and the clinical management of the mutation carriers.

## Conflict of Interest

The authors declared no conflict of interest.

## Supporting information


**Table S1.** Breast/ovarian cancer families investigated for BRCA1/2 mutations.

**Table S2.** Features of the BRCAX families.

**Table S3.** Mutated cancer tissues LOH.
