# Supplementary material for: Optimizing the identification of risk‐relevant mutations by multigene panel testing in selected hereditary breast/ovarian cancer families
Source: Cancer Med. 2017 Dec 22;7(1):46–55. doi: 10.1002/cam4.1251 (PMC5773970; doi:10.1002/cam4.1251)
Supplement: Supplementary file 1 — Table S1. Breast/ovarian cancer families investigated for BRCA1/2 mutations. [file CAM4-7-46-s001.doc]

|  | **No. of families**  **(%)** | **No. of mutations (mutation rate)** | **BRCA1**  **%** | **BRCA2**  **%** | **T** |
| --- | --- | --- | --- | --- | --- |
| **BC** | 266 (72%) | 36 (13%) | 16 (6%) | 20 (7,5%) | 36 |
| **BOC** | 101 (28%) | 61 (60%) | 46 (45%) | 15 (15%) | 61 |
| **Tot** | 367 | 97 (26%) | **62** | **35** | 97 |

**Supplementary TABLE 1.** Breast/ovarian cancer families investigated for BRCA1/2 mutations

Abbreviations: BC, breast cancer-only families; BOC, breast and ovarian cancer families
