# Supplementary material for: Optimizing the identification of risk‐relevant mutations by multigene panel testing in selected hereditary breast/ovarian cancer families
Source: Cancer Med. 2017 Dec 22;7(1):46–55. doi: 10.1002/cam4.1251 (PMC5773970; doi:10.1002/cam4.1251)
Supplement: Supplementary file 2 — Table S2. Features of the BRCAX families. [file CAM4-7-46-s002.doc]

**Supplementary TABLE 2.** Features of the BRCAX families

| **Family ID** | **Syndrome** | **Cancer spectrum**  **(age)** | **Proband cancer**  **(age)** | **BRCApro5**  **%** | **Previous characterization** |
| --- | --- | --- | --- | --- | --- |
| BR17 | BC | BC: (33); BC: (49); bil BC: (49; 56); bil BC: (44; 44) | bil BC: (49; 56) | 97 | *BRCA1/2* seq + MLPA |
| BR166 | BC | BC: (38); BC: (60); bil BC: (50; 50)  bil BC: (51; 51); bil BC: (52; 52)  bil BC: (55; 55); bil BC: (58; 58) | BC: (38) | 95 | *BRCA1/2* seq + MLPA;  *PALB2* seq |
| BR501 | BC | BC: (25); BC: (43); BC: (50); BC: (55)  bil BC: (55; 56); BC: (60) | BC: (25) | 94 | *BRCA1/2* seq |
| BR362 | BC | bil BC: (49; 49); bil BC: (30; 37); BC: (58)  ≥ 3th degree: Leu: (3) | bil BC: (49) | 93 | *BRCA1/2* seq + MLPA |
| BR409 | BC | BC: (40); bil BC: (30; 32); bil BC: (63; 63);  EC: (50); UC: (75)  ≥ 3th degree: Neuroblastoma: (2) | bil BC: (30; 32) | 91 | *BRCA1/2* seq |
| BR225 | BC | BC: (42); BC: (30), NHL: (70); MB: (64);  bil BC: (40, 52), Thyr: (45), KC: (64);  GC: (65); GC: (67); GC: (71); CC: (80) | BC: (42) | 91 | *BRCA1/2* seq + MLPA; *PALB2* seq |
| BR278 | BC | BC: (35); bil BC: (35; 35) | bil BC: (35; 35) | 87 | *BRCA1/2* seq + MLPA |
| BR107 | BC | BC: (47); BC: (49); BC: (50);  bil BC: (43; 43);  ≥ 3th degree: BC: (46) | BC: (47) | 82 | *BRCA1/2* seq + MLPA; *PALB2* seq |
| BR208 | BC | BC: (40); BC: (38); BC: (38); BC: (60);  BC: (60); BC: (60); PrC: (69); PrC: (70);  ≥ 3th degree: BC: (38); BC: (48); Mel: (44) | BC: (40) | 82 | *BRCA1/2* seq + MLPA;  *PALB2* seq |
| BR60 | BC | BC: (70), LC: (79); BC: (40); BC: (43); BC: (47); bil BC: (48; 53);  EC: (51);  ≥ 3th degree: BC: (52); Thyr: (30) | BC: (70), LC: (79) | 80 | *BRCA1/2* seq + MLPA |
| BR404 | BOC | bil BC: (73; 73); OC: (43); OC: (46);  OC: (55), CC: (47); PrC: (60) | bil BC: (73; 73) | 75 | *BRCA1/2* seq |
| BR336 | BOC | bil BC: (44; 44); OC: (65);  EC: (81); PrC: (78); PrC: (81) | bil BC: (44; 44) | 66 | *BRCA1/2* seq + MLPA |
| BR302 | BC | bil BC: (38; 49); bil BC: (64; 65) | bil BC: (38; 49) | 64 | *BRCA1/2* seq + MLPA |
| BR377 | BC | BC: (39); BC: (42), PC: (47); BC: (68);  GC: (81); KC: (44) | BC: (39) | 64 | *BRCA1/2* seq + MLPA |
| BR232 | BC | BC: (26); BC: (40), CNS: (75); BC: (50); BC: (50); CC: (52) | BC: (26) | 54 | *BRCA1/2* seq + MLPA; *PALB2* seq |
| BR273 | BC | BC: (48); BC: (53); bil BC: (49; 53);  H&N: (65) | BC: (48) | 50 | *BRCA1/2* seq + MLPA |
| BR194 | BC | BC: (55); bil BC: (43; 60); bil BC: (61; 63);  HCC: (51) | bil BC: (43; 60) | 50 | *BRCA1/2* seq + MLPA; *PALB2* seq |
| BR48 | BC | bil BC: (36; 41); BC: (46); LC: (85) | bil BC: (36; 41) | 47 | *BRCA1/2* seq + MLPA; *PALB2* seq |
| BR38 | BOC | OC: (41), BC: (48); BC: (59); LC: (59) | OC: (41), BC: (48) | 36 | *BRCA1/2* seq + MLPA |
| BR494 | BC | bil BC: (38); BC: (59);  ≥ 3th degree: BC: (42); BC: (44) | bil BC: (38) | 26 | *BRCA1/2* seq |

Abbreviations: BC, breast cancer; bil BC, bilateral breast cancer; MB, male breast; OC, ovarian cancer; EC, endometrial cancer; GC, gastric cancer; CC, colon cancer; Mel, melanoma; PC, peritoneal carcinosis; KC, Kidney cancer; LC, lung cancer; PrC, prostatic cancer; NHL, non Hodgkin lymphoma; CNS, central nervous system cancer; Leu, leukemia; Thyr, Thyroid cancer; H&N, head&neck; HCC, hepatocarcinoma; UC, unknown cancer
