# Supplementary material for: Optimizing the identification of risk‐relevant mutations by multigene panel testing in selected hereditary breast/ovarian cancer families
Source: Cancer Med. 2017 Dec 22;7(1):46–55. doi: 10.1002/cam4.1251 (PMC5773970; doi:10.1002/cam4.1251)
Supplement: Supplementary file 3 — Table S3. Mutated cancer tissues LOH. [file CAM4-7-46-s003.doc]

**Supplementary TABLE 3. Mutated cancer tissues LOH**

| **Family ID** | | **Gene** | **Sample histotype** | **LOH** |
| --- | --- | --- | --- | --- |
| **BR208** | proband | *ATM* | NT | Htz |
|  | proband | *ATM* | LIN/DIN | Htz |
|  | proband | *ATM* | BC-IDC | Htz |
| **BR225** | proband | *ATM* | BC-IDC | Htz |
|  | father | *ATM* | MB-IDC | Htz |
|  | Rel1 | *ATM* | CRC | Htz |
|  | Rel2 | *ATM* | NHL-LN | Htz |
| **BR404** | proband | *BRCA1* | BC-IDC | LOH |
|  | Rel1 | *BRCA1* | HGSOC | LOH |
|  | Rel2 | *BRCA1* | HGSOC | LOH |
| **BR501** | Rel1 | *CHEK2* | BC-IDC | LOH |

Abbreviations: NT, normal tissue; BC-IDC, breast cancer-invasive ductal carcinoma; MB-IDC, male breast cancer-invasive ductal carcinoma; DIN, Ductal Intraepithelial Neoplasia; LIN, Lobular Intraepithelial Neoplasia; HGSOC, high-grade serous ovarian cancer; CRC, Colorectal Cancer; NHL-LN, NHL affected lymph-node; Htz, Heterozygous
